# Addressing barriers to the conduct and application of research in complementary and alternative medicine: a scoping review

**DOI:** 10.1186/s12906-021-03371-6

**Published:** 2021-07-15

**Authors:** Yasamin Veziari, Saravana Kumar, Matthew Leach

**Affiliations:** 1 UniSA Allied Health and Human Performance, University of South Australia, North Terrace, Adelaide, SA 5000 Australia; 2 National Centre for Naturopathic Medicine, Southern Cross University, Military Road, East Lismore, NSW 2480 Australia

**Keywords:** Complementary and alternative medicine, Complementary therapies, Scoping review, Evidence-based practice, Evidence-based medicine, Knowledge translation, Knowledge transfer, Barriers, Enablers, Facilitators, Strategies, Conduct of research, Application of research

## Abstract

**Background:**

Over the past few decades, the popularity of complementary and alternative medicine (CAM) has grown considerably and along with it, scrutiny regarding its evidence base. While this is to be expected, and is in line with other health disciplines, research in CAM is confronted by numerous obstacles. This scoping review aims to identify and report the strategies implemented to address barriers to the conduct and application of research in CAM.

**Methods:**

The scoping review was undertaken using the Arksey and O’Malley framework. The search was conducted using MEDLINE, EMBASE, EMCARE, ERIC, Scopus, Web of Science, The Cochrane Library, JBI and the grey literature. Two reviewers independently screened the records, following which data extraction was completed for the included studies. Descriptive synthesis was used to summarise the data.

**Results:**

Of the 7945 records identified, 15 studies met the inclusion criteria. Using the oBSTACLES instrument as a framework, the included studies reported diverse strategies to address barriers to the conduct and application of research in CAM. All included studies reported the use of educational strategies and collaborative initiatives with CAM stakeholders, including targeted funding, to address a range of barriers.

**Conclusions:**

While the importance of addressing barriers to the conduct and application of research in CAM has been recognised, to date, much of the focus has been limited to initiatives originating from a handful of jurisdictions, for a small group of CAM disciplines, and addressing few barriers. Myriad barriers continue to persist, which will require concerted effort and collaboration across a range of CAM stakeholders and across multiple sectors. Further research can contribute to the evidence base on how best to address these barriers to promote the conduct and application of research in CAM.

**Supplementary Information:**

The online version contains supplementary material available at 10.1186/s12906-021-03371-6.

## Background

Evidence-based practice (EBP) is an approach whereby healthcare decisions are based on the best available, current, relevant and valid evidence and where there is continual evolvement of techniques, procedures and policies. The intent of EBP is to reduce variations in care, increase patient safety and improve patient outcomes [1]. Over the past few decades, the original definition of evidence-based practice [2] has evolved in the *“*integration of the production and the application of research evidence*”* [3]. There is increasing pressure within all health sectors to generate [4], implement and evaluate evidence [5, 6], in combination with a patient’s preferences and needs. Even though engagement in EBP may lead to greater professional satisfaction, EBP is not consistently used across all disciplines [7–9].

In 2009, the National Academy of Medicine’s Roundtable Conference on Evidence-based Medicine set an ambitious goal that 90% of clinical decisions will be based on evidence by 2020 [10]; unfortunately, this goal has yet to be achieved [11]. This is partly because barriers to evidence-based practice (EBP) uptake continue to exist in all areas of healthcare [12–14] including barriers to conducting research for the generation of evidence [15–17] as well as barriers to applying evidence into practice [13, 14]. To improve EBP uptake and in turn, the quality of health care, strategies addressing existing barriers are constantly being examined. There are also calls to improve health service delivery through innovation [18, 19] and evidence [4, 20], and to address barriers to evidence-based practices within conventional and allied healthcare [21–23]. CAM too is being subjected to similar evidence rules [24–26].

“Complementary and alternative medicine”, is an umbrella term capturing a diverse group of therapies not considered part of the conventional medical system [27]. CAM practices can be divided into five broad categories including, *Mind-body medicine, Whole medical systems, Energy medicine*, *Biologically based practices,* and *Manipulative/Body-based practices* [28]*.* This group of health-care practices use interventions and approaches that promote the innate healing ability of the body while retaining a core focus on individuality, holism, education, and disease prevention.

CAM use is growing internationally [29, 30]. Many factors can be attributed to the rising interest in CAM, including the move towards holistic well-being, the recognition of limitations of conventional medicine, increasing healthcare costs and the growing discourse on the important contribution of CAM [30]. The growing popularity of CAM has been paralleled by increased scrutiny of the evidence-base of the field, with calls for more research and critical appraisal of the evidence underpinning CAM [31]. Corresponding to calls for more CAM research is recognition of the numerous barriers to conducting rigorous research in CAM [32–35] and the challenges in translating this evidence into practice [36].

The EBP movement has placed considerable pressure on the field of CAM, which historically has relied heavily on traditional, experiential evidence [34]. The tension between traditional versus scientific evidence continues to persist in CAM, for which there may be a number of explanations. A recent systematic review [35] comprehensively mapped a range of obstacles to engaging with research within the field of CAM. These obstacles were divided into two broad categories: (1) Barriers to the *conduct* of research (i.e. evidence generation), and (2) Barriers to the *application* of research (i.e. evidence utilisation). The review highlighted the multifactorial and complex nature of these barriers, and the need for a comprehensive, systematic, and targeted approach to addressing these barriers.

To date, there has been no synthesis of strategies aimed at overcoming barriers to the conduct and application of research in CAM. This review aims to address this knowledge gap, and in doing so, may identify potential strategies that could help improve EBP uptake in CAM.

## Methods

### Study design

Scoping reviews are a relatively new but an increasingly common approach for mapping broad topics [37, 38]. A scoping review methodology was selected over other review methodologies as it can comprehensively map evidence across a range of study designs in a broader area of interest, and identify knowledge gaps to help inform future research practice, systematic reviews or programs/policy [37, 38]. The protocol for this scoping review adheres to the PRISMA-ScR guidelines [39] and is informed by a related scoping review in the field of chiropractic [40]. This scoping review also follows established frameworks in the conduct and reporting of scoping reviews, including those reported by Arksey and O’Malley [41], advanced by Levac and colleagues [42], and published by the Joanna Briggs Institute (JBI) [43].

### Identifying the research question

The scoping review aimed to answer the following research question: *“What enabling strategies have been implemented to address barriers to the conduct and application of research in complementary and alternative medicine?”*

### Identification of relevant studies

A search strategy was developed for MEDLINE (Appendix 1. MEDLINE search strategy) with guidance from an academic librarian. The search strategy was modified for use in other databases including EMBASE, EMCARE, ERIC, Scopus, Web of Science, The Cochrane Library, and the Joanna Briggs Institute EBP database. Google Scholar, the Google search engine (up to the first ten pages) [44] and MedNar were searched to identify relevant grey literature, blogs and reports. ProQuest and Trove were also searched to identify theses/dissertations and conference abstracts or proceedings. Reference lists of included studies were scanned to ensure no relevant studies were missed. The reviewer also intended to contact authors of primary studies or reviews for further information, if required. Publications were restricted to those published only in the English language. No limits were applied to the publication date. Selected literature was exported and saved on EndNote™, screened for duplicates, and exported to Covidence™ for a second screening of duplicates and eligibility screening. The search was operationalised between January and May 2019 and updated on 1st June 2021.

### Study selection

#### Inclusion criteria

Primary studies (quantitative and qualitative) reporting the application and evaluation of any enabling strategy/intervention aimed at addressing barriers to the conduct or application of research within CAM, were eligible for inclusion. This included both published and unpublished studies.

#### Exclusion criteria

Opinion articles, discussion papers and reviews were not eligible for inclusion. Also excluded from the review were studies focusing on CAM products, treatments or remedies, vendors and manufacturers of CAM products, integrative medicine or conventional medicine practitioners and bio-medical researchers. Studies examining knowledge of CAM, attitudes towards CAM or the effectiveness of CAM were also excluded.

#### Screening

Two researchers independently screened the title and abstract of all retrieved studies to determine eligibility against the review selection criteria. Studies considered potentially eligible for inclusion were screened in full text by two researchers, independently. Conflicts between researcher decisions were discussed, and if disagreement persisted, decisions were resolved by consulting a third researcher.

### Data extraction

A customised data extraction form was developed for the review [45]. The data extraction form was piloted by two researchers using a sample of one included article. Duplicated and irrelevant variables were removed after pilot testing. The data extraction form was informed by the aim of the review, research team expertise, and literature on barriers to research conduct or application. Items included in the form were author(s), year, country, design/method, objectives, CAM disciplines, participants/sample size, enabling strategies (concept/context, characteristics, funding/grants, outcome domains measured), results (attitudes, skills, knowledge, competencies), barriers addressed (conduct/application of research), study limitations and future recommendations. Data were extracted by YV and SK, and verified by ML. In accordance with scoping review guidelines, included studies were not appraised [37].

### Collating, summarising and reporting

Data extracted from each included article were collated and reviewed by the research team. Any discrepancies in extracted data were discussed until consensus was reached. Data were then synthesized in narrative form. The “BarrierS To the Application and Conduct of rEsearch” (oBSTACLES) instrument [46] was used as a guide to classify barriers (i.e. conduct or application of research) addressed by the enabling strategies. The oBSTACLES instrument was selected as it (a) maps barriers to both the conduct and application of research [35], (b) is published and psychometrically tested [46, 47], and (c) maps the continuum of evidence from conduct to application. The results of this scoping review were also reported in the Preferred Reporting Items for Systematic Reviews and Meta-Analyses extension for scoping reviews [39, 48].

## Results

### Search results

**Fig. 1:**
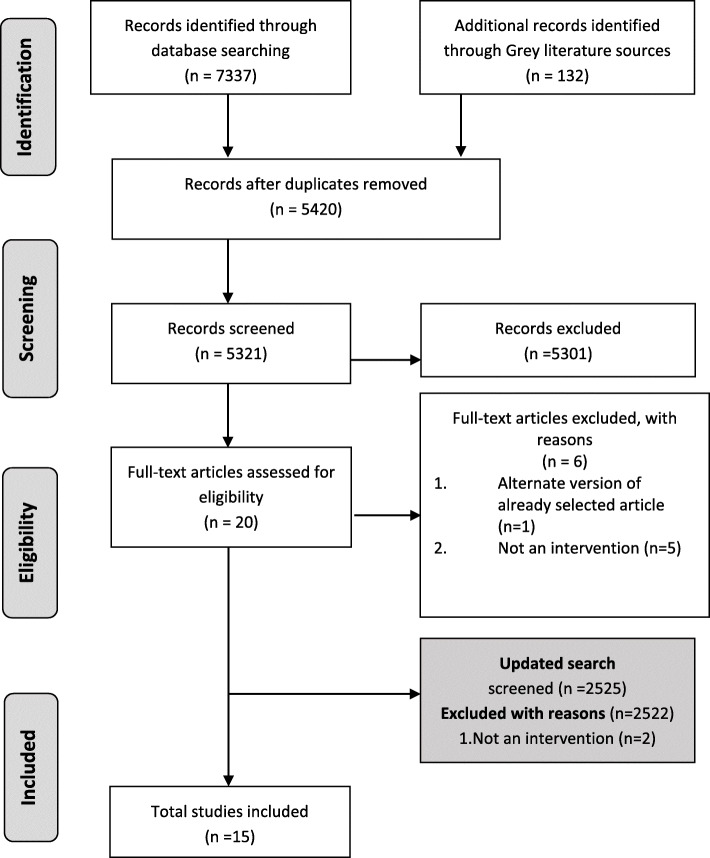
Flow chart of the study selection process

The initial search identified 7601 records (Fig. 1). After the removal of duplicates, a total of 5321 titles and abstracts were screened, of which 5301 did not meet the inclusion criteria. The 20 remaining records were screened in full text; 6 records were excluded as they were either duplicate records (*n* = 1) or did not report a strategy/intervention (*n* = 5). The remaining 14 studies were included in this review. An updated search was conducted on 1st June 2021, which resulted in 2525 citations. After title and abstract screening, 3 articles proceed to full text screening. Full text screening resulted in exclusion of 2 articles (as they did not report intervention strategies) and inclusion of 1 additional article. Therefore, the final number of included studies in this review was 15.

### Description of the included studies

The fifteen included studies [49–63] were published between the years 2008 and 2019 (Table 1). Most studies were conducted in the USA (*n* = 11; 73%) [49, 50, 52–55, 58–62], followed by Australia (*n* = 2; 13%) [56, 57], and China (*n* = 2; 13%) [51, 63]. All studies were undertaken in the educational sector. Myriad study designs were reported, including survey (*n* = 5) [49, 56, 59, 61, 62], descriptive (*n* = 2) [53, 55], multi-method (*n* = 2) [57, 60], action research (*n* = 1) [51], prospective cohort (*n* = 1) [50], pre-post (*n* = 2) [54, 63], qualitative (*n* = 1) [52], and exploratory randomised trial (*n* = 1) [58].

**Table 1:** Characteristics of included studies

Author (Year)	Methodology utilised	Country	Participants sample size (n)	EBP Barriers addressed	Funding (Y/N)	Interventions
Allen et al. (2011) [49]	Survey	USA	Faculty (*n* = 11)	Lack of EBP approaches among CAM practitioners	Y	University developed *EBP course for Natural & Chinese medicine faculty
Caldwell et al. (2018) [51]	Action research (survey, focus groups, diary notes, field notes)	China	Teachers, Assistants (*n* = 25) & students(*n* = 86)	Lack of research education and training	Y	International collaboration to assess initial *EBP workshop to help in the redesign
Cramer et al. (2015) [59]	Survey	USA	Principle investigators from CAM and traditional research-intensive institutions (n-19)	Lack of research culture in CAM institutions; lack of collaboration between CAM and non-CAM institutions and faculty	Y	CAM Faculty & *TRI collaboration (seminars, mentorship joint development of courses, *EBP resources, and consulting)
Evans et al. (2011) [53]	Descriptive	USA	Faculty and students	Lack of research education and training among CAM practitioners	Y	CAM & *TRI collaborated faculty development research program
Haas et al. (2012) [50]	Prospective cohort (exam scores, questionnaire)	USA	Students (*n* = 370)	Poor knowledge, attitude, behaviour and skills in EBP	Y	CAM College & *TRI partnership to incorporate *EBP curriculum in existing 4-year program
Laird et al. (2010) [54]	Pre and post	USA	Course directors (*n* = 34)	Lack of EBP learning in course content	Y	*EBP workshops series for faculty
Long et al. (2014) [62]	Survey	USA	Program directors of CAM academic institutions (*n* = 9)	Lack of research expertise, literacy and evidence-based practice among CAM faculty	Y	*EBP literacy & training (e.g. workshops, seminars, online resources, short courses, intensive multiday training programs)
McCarty et al. (2011) [52]	Qualitative (Focus group, Interviews)	USA	Faculty (n = 9)	Lack of EBP in CAM education and practice	Y	Clinical Exchange program between CAM school and *TRI
Schneider et al. (2016) [58]	Exploratory randomised trial (survey)	USA	Practitioners (*n* = 293)	Lack of online EBP distance-learning	Y	Online *EBP course and booster lessons
Steel, Adams, Sibbritt (2014) [57]	Multi-method (Audit, survey)	Australia	Practitioners (*n* = 1306)	Disconnect between researchers and practitioners	Y	Establish a protocol for a multi-modality *PBRN
Steel, et al. (2018) [56]	Survey	Australia	Practitioners (*n* = 764)	Disconnect between researchers and practitioners	Y	Multi-modality, national *PBRN
Sullivan, Furner & Cramer (2013) [60]	Multi-method (summaries, semi-structured interviews, surveys)	USA	Pre-doctoral CAM Students (*n* = 6)	Need for translational, interdisciplinary, and integrative research in CAM	Y	*TRI mentored research program for CAM institution
Wayne et al. (2008) [55]	Descriptive	USA	Faculty, staff, alumni, students	Lack of research education and training in CAM institutions	Y	Training and research literacy collaboration between CAM school & Medical School
Wong et al. (2019) [63]	Pre and post	China	Students (*n* = 59)	Lack of EBP in practice	Y	Face-to-face 3-day workshop
Zwickey et al. (2014) [61]	Survey	USA	CAM colleges (n = 9)	Lack of research education and training in EBP	Y	Curricular revision of research literacy teaching

*EBP Evidence-based practice, *PBRN Practitioner-based research network, *TRI Traditional research-intensive

All studies (100%) were undertaken in the educational sector. Ten studies (66.6%) reported the provision of funding by the National Institute of Health (NCCAM) to improve research conduct or application within CAM institutions and practice [49, 50, 52, 53, 55, 58–62]. The fifteen included studies targeted twenty-one CAM disciplines (i.e. Acupuncture, Aromatherapy, Ayurveda, Bowen therapy, Classical Chinese medicine, Chinese Herbal medicine, Chiropractic, Counselling, Homeopathy, Kinesiology, Massage therapy, Musculoskeletal therapy, Myotherapy, Naturopathy, Nutrition (non-dietetic), Osteopathy, Oriental medicine, Reflexology, Traditional Chinese medicine, Western Herbalism and Yoga). Seven studies (46.6%) focused on a single CAM discipline [50–52, 54, 55, 58], and eight studies (53%) focused on multiple CAM disciplines [49, 53, 56, 57, 59–62]. Disciplines represented the most in the included studies were Chiropractic (*n* = 7 studies), aturopathy (*n* = 7 studies), Acupuncture (*n* = 6 studies), Massage therapy (*n* = 5 studies), Osteopathy (*n* = 4 studies), Chinese Herbal Medicine (*n* = 4 studies), and Nutrition (*n* = 3 studies). Aromatherapy, Ayurveda, Bowen Therapy, Homeopathy, Kinesiology, Myotherapy, Musculoskeletal therapy, Oriental medicine, Reflexology, Traditional Chinese medicine, Western Herbalism and Yoga were represented in two studies each. Counselling, and Classical Chinese medicine were each reported in a single study.

### Description of enabling strategies

All fifteen studies reported enabling strategies that focused on both education and collaborative activities. Eleven studies (73%) reported education strategies [49–51, 53–55, 58, 60–63], and eleven studies (73%) [49–57, 59, 60] reported on the formation of networks or collaborations. Among the eleven studies that reported collaborations, nine studies (60%) reported collaborations between CAM institutions (schools, colleges) and traditional research intensive (TRI) non-CAM institutions (i.e. conventional universities, medical schools) [49–55, 59, 60], and two studies reported on the formation of a practice-based research network (PBRN) [56, 57].

### Description of barriers addressed

**Fig. 2:**
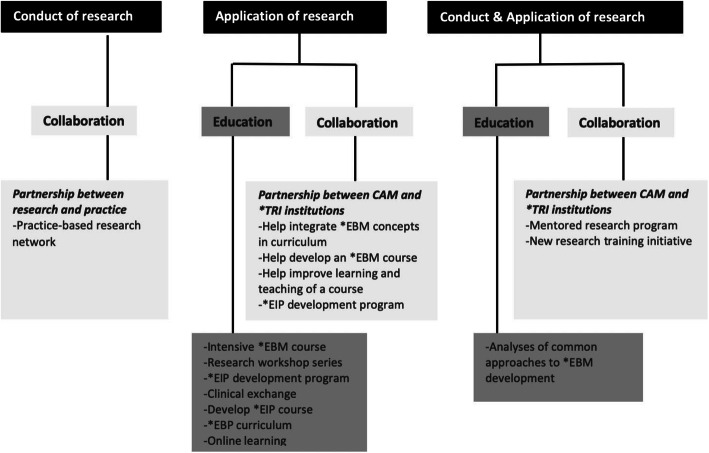
Enabling strategies that address the three barrier groups. *EIP = Evidence informed practice; *EBM = Evidence based medicine; *EBP = Evidence-based practice; *TR I = Traditional research-intensive

Informed by the oBSTACLES instrument, the enabling strategies reported in the included studies were categorised into three distinct groups, including those that addressed: (a) barriers to the conduct of research, (b) barriers to the application of research, and (c) barriers to both the conduct and application of research. Figure 2 summarises the enabling strategies captured within each of these groups. A supplementary data extraction file is also provided.

#### Strategies addressing barriers to the conduct of research

Two studies (13%) [56, 57] reported enabling strategies that solely addressed barriers to the conduct of research. Both studies reported the establishment of a practice-based research network (PBRN). While neither study reported the impact of the PBRN, the authors indicated that the PBRN aimed to provide infrastructure for researchers to engage with practitioners in grass-roots clinical practice to facilitate research inquiry [57].

### Strategies addressing barriers to the application of research

Eight studies (53%) [49–54, 58, 63] reported enabling strategies that addressed barriers to the application of research in CAM practice. All eight studies used education as an enabling strategy that targeted CAM faculty, staff, students and/or practitioners. Six studies [49–54] reported on collaborative education initiatives between CAM educational institutions and TRI institutions, such as the implementation of an intensive short course in EBM [49], research workshops [54, 63], an evidence-informed practice (EIP) development program [53], the integration of EBP curriculum into an existing program [50], improvement of a previously developed course [51], and clinical exchange between CAM faculty and an allopathic clinic [52]. One study, not linked to any collaborative activity, used a standalone online distance education program to teach practitioners about the principles of EBP [58].

#### Outcomes for faculty

Five studies that addressed barriers to the application of research reported outcomes for faculty. The educational and collaborative strategies reportedly improved faculty attitude, such as commitments to enhancing skills, using EBM in teaching [49], adopting interactive teaching methods [51], improved confidence [54], change in culture [49], appreciation of the exposure to clinical experiences outside the usual naturopathic scope [49], affirmation of naturopathic profession/training [52], and the value of observations of clinical resources in a university setting [52]. Changes in participant knowledge and skills were also reported including improvements in practical skills [49], understanding of EBM [54], and the ability to teach critical appraisal and apply it to patient care [54]. Furthermore, the initiatives appeared to forge new relationships with the clinical exchange experience [52] being viewed as a favourable way to help integrate EBM into CAM clinical teaching. There was also an impact on teaching and learning. Resources, including improvements to existing teaching tools [51, 52], development of new EBP courses [53], and the creation of a CAM school-specific library modelled after the collaborating university library [52].

#### Outcomes for students

Three studies addressing barriers to the application of research reported outcomes for students. The educational strategies employed in these studies appeared to enhance student attitudes [50, 51], confidence [63], knowledge, self-assessed skills and behaviours regarding the utilisation of EBP [50, 63]. One study [63] reported that despite the use of an educational strategy involving a 3-day workshop, subjective norms about EBP did not change.

#### Outcomes for practitioners

One study addressing barriers to the application of research reported outcomes for practitioners. Authors of a single trial reported a modest improvement in practitioner attitude and skills in EBP, but not in EBP use, among practitioners assigned to the online educational strategy compared with those allocated to waitlist control [58].

### Strategies addressing barriers to the conduct and application of research

Five studies (33%) [55, 59–62] reported enabling strategies that addressed barriers to the conduct and application of research. Of these, three studies reported the use of both educational and collaborative strategies including cross-institutional research training [55], a research mentorship for CAM students [60] and evaluating best practice models for implementing curricular and culture change [61]. One study examined the approaches used to develop faculty expertise in research literacy and EBP [62]. One study reported the impact of collaborative strategies between CAM and TRI institutions to increase the quality and quantity of research content and curricula [59].

#### Outcomes for faculty and staff

Four studies addressing barriers to the conduct and application of research reported outcomes for faculty and staff. The enabling strategies reportedly enhanced faculty attitudes, confidence, and skills in EBP and research [55, 59, 62], as well as faculty and staff research experience [59]. Incentives such as providing credit for continuing education [55, 62], allowing time for participation in research-related conferences and seminars and creating opportunities to apply for small grants to attend research-related seminars and conferences helped to reinforce the idea that research should inform practice [62]. Improved access to resources and research training [62] was also reported. One study described improvements in research output [55], with the strategy contributing toward the publication of more than 25-peer reviewed papers [55]. Cross-institutional collaborations reportedly helped CAM institutions capitalise on resources provided by conventional partners, enabled CAM faculty/staff to enroll in university clinics and research training programs, developed instructional approaches in research literacy and EBP programs, developed assessment tools and strategies to evaluate faculty development, and provided mentorship opportunities [62].

#### Outcomes for students

Student outcomes were reported in three studies addressing barriers to the conduct and application of research. The use of both educational and collaborative strategies were reported to improve student learning, knowledge acquisition, application and demonstration of competence in research [61], participation in research studies (including increased participation in masters and doctoral programs) [60, 61], confidence in undertaking independent studies [55], developing research clubs [61], and including research as a significant component of their career [55].

#### Outcomes for institutions

Four studies addressing barriers to the conduct and application of research reported outcomes for institutions. The use of education and training initiatives, along with cross-institutional collaborations, appeared to transform a “vocational” institution into an “academic” institution [55], elevated EBP content in curricula and clinical training [55, 61, 62], increased the number of research faculty appointments with doctoral degrees [55], and created new programs to support faculty development in medical education [62] and Integrative Medicine research [61]. Cross-institutional collaborations also reportedly improved the culture and relationships between CAM and clinical science faculties [59, 61], helped devlop a greater understanding of similarities and differences between healthcare disciplines and paradigms, and improved perceptions of the viability of future collaborations [59]. One study also indicated that as a result of the strategy, librarians emerged as leaders in supporting understanding and use of EBM resources, and facilitating the development and implementation of systems for teaching EBM content [61].

## Discussion

This is the first known synthesis of evidence of strategies aimed at addressing barriers to the conduct and application of research in CAM. By doing so, this review addresses an important knowledge gap in the literature. While there have been some concerted efforts to overcome these barriers, this review found the evidence to be limited to a handful of jurisdictions (e.g., United States of America), a small number of CAM disciplines (e.g., chiropractic, naturopathy, and acupuncture), and addressed few barriers (e.g., skill development, collaborative and targeted funding opportunities).

Education was the most frequently used strategy to address gaps in knowledge, participation, attitudes, and skills as a means of improving CAM practitioner, faculty, staff, and student engagement in research. The use of educational strategies to promote engagement with research is not unique to CAM and has been reported widely across a range of health disciplines. For example, in medicine [64], and in allied health [65], numerous training programs have been used to improve research literacy. There is considerable research relating to using education to address barriers to incorporate EBM principles and practices in healthcare [21, 66–69]. Educational strategies, targeted at institutions that train CAM practitioners, may assist in developing future CAM workforce that is skilled, and confident to engage in research. For those in clinical practice, embedding research and EBP training in continuing professional development requirements may provide an incentive to upskill in these areas [36, 70]. However, while such initiatives have been successfully utilised in medicine and allied health [71, 72], they may not be necessarily successful in CAM.

The reluctance of many CAM disciplines to engage in EBP or research may be attributed to epistemological [73] and philosophical [74] differences between CAM and bio-medical disciplines. For example, CAM approaches towards disease are often reported to be incompatible with standardized research protocols like randomised controlled trials [75] which rarely reflect the individualised, multi-modality delivery of CAM interventions [76]. As a result, what may be considered robust research and evidence by biomedical disciplines, may not be shared by CAM disciplines [73, 74, 76–80]. Other challenges impacting CAM practitioner engagements include barriers to training (i.e. lack of dedicated research training for CAM disciplines) [81], in clinical practice (i.e lack of incentives and time, financial disincentives and the need to ensure financial survivability [82]) and the lack of a research culture [35]. Notwithstanding, these barriers are shared across several health disciplines, including medical [17, 83] and allied health [84–90].

As a means of addressing barriers to engagement with research, many of the included studies utilised collaborative approaches, such as building relationships between TRI institutions and CAM stakeholders and creating a PBRN. Linking with TRI institutions could be a worthwhile strategy as these institutions have long incorporated research and evidence-based practice principles within their curricula [64, 91, 92]. These learnings and experiences could be used by CAM stakeholders to develop a CAM workforce that has the knowledge, skills, and competencies to engage with research.

The use of PBRNs has been shown to be another effective strategy for improving the conduct and application of research as it is a mature collaborative effort that can facilitate a research culture for practitioners [93], as well as provide a necessary first-step to EBP [82]. PBRNs can also serve as ideal environments to increase understanding of barriers to professional behaviour change [40]. This is critical as PBRNs can provide useful insights into barriers that may confront a workforce when engaging with research at the frontline of clinicial practice. This can inform development of practitioner-driven strateiges to support “bench to behaviour” [94].

The important role of funding, the impact of the lack thereof, has been widely reported in the literature [95, 96]. Funding can influence the production of knowledge [97, 98], and this review found many of the strategies reported in this review were developed with the support of targeted funding opportunities for CAM researchers and practitioners; without which, these developments may not have been possible. While such targeted approaches have reported benefits [99, 100], they have been confined to limited jurisdictions and thus require wider implementation and evaluation.

### Strengths, limitations, and recommendations

This review has several strengths. This scoping review was underpinned by rigorous and transparent methods and followed best practices in the conduct and reporting of a scoping review. The review protocol was devised and reviewed by members of a research team with significant expertise in knowledge synthesis and review methods, and the search strategy was independently validated by an academic librarian. The inclusion of both quantitative and qualitative study designs added to the analytical breadth and depth of this review.

However, as with any research, this review too has limitations. First, searching CAM-related literature can be challenging due to the diversity of professions classified as CAM; further, not all CAM literature are published in indexed journals. Second, this scoping review was limited to studies published in the English language, therefore, it is possible that relevant studies and insights may have been missed.

While several barriers to the conduct and application of research in CAM have been identified [46], the review identified studies that targeted only a handful of these barriers. Collectively, these barriers could be categorised into those associated with knowledge and skills (e.g. limited knowledge and skills to apply research evidence into practice), capacity (e.g. limited opportunities for CAM undergraduate students to contribute to CAM research), collaborative opportunities (e.g. limited collaboration between CAM researchers and other health researchers) and funding. The narrow range of barriers addressed by initiatives to date is a limitation of the current knowledge base; notwithstanding, it does highlight potential opportunities for future research (i.e. addressing other barriers to the conduct and application of research in CAM). These research initiatives could explore innovative strategies to address CAM-centric barriers to research (such as customised educational programs) or build on strategies that have demonstrated impact elsewhere (such as collaborative approaches with TRI institutions).

## Conclusion

Despite the growing popularity of CAM and wide-spread recognition of research to inform CAM practices, there continue to persist numerous barriers to the conduct and application of research in CAM. While this has been recognized, much of the focus to date has been limited to initiatives originating from a handful of jurisdictions and mainly for a small group of CAM disciplines; these initiatives also target few barriers. While research in this field is encouraging, myriad barriers continue to persist, and the effectiveness of these initiatives warrants further examination. Addressing these barriers, will require concerted effort and collaboration by a range of CAM stakeholders and across multiple sectors. Further research can contribute to the evidence base on how best to address these barriers to promote the conduct and application of research in CAM.

### Supplementary Information

**Additional file 1.**

## Data Availability

All data generated or analysed during this study are included in this published article.

## Appendix 1

### Medline search

1-complementary therapies/

2-acupuncture therapy/

3-acupuncture analgesia/

4-acupuncture, ear/

5-homeopathy/

6-Aromatherapy/

7-Medicine, Ayurvedic/

8-medicine, traditional/

9-mind-body therapies/

10-yoga/

11-naturopathy/

12-Massage/

13-Herbal Medicine/

14-ACUPUNCTURE/

15-Manipulation, Chiropractic/

16-Manipulation, Osteopathic/

17-KINESIOLOGY, APPLIED/

18-Medicine, Chinese Traditional/

19-NUTRITIONISTS/

20-OSTEOPATHIC PHYSICIANS/

21-(Complementary medicine? or alternative medicine? or chinese medicine? or traditional medicine? or alternative therap* or complementary therap* or traditional therap* or acupunct* or homeopath* or mind-body therap* or mind body therap* or yoga or naturopath* or massage? or massaging or herbal medicine? or herbalism or osteopath* or chiropract* or TCAM? or ayurved* or hindu medicine? or siddha medicine? or myotherapy* or bowen?? therap* or aromatherap* or aroma therap* or reflexolog* or bodywork or bodyworks or kinesiolog* or nutrition or clinical nutritionist?).mp. [mp = title, abstract, heading word, drug trade name, original title, device manufacturer, drug manufacturer, device trade name, keyword, floating subheading word, candidate term word].

22-or/1–21

23-Evidence-Based Practice/

24-(evidence? base? or evidence?-base? or EBP).mp. [mp = title, abstract, heading word, drug trade name, original title, device manufacturer, drug manufacturer, device trade name, keyword, floating subheading word, candidate term word]

25-Policy/

26-Organizational policy/

27-(policy or policies).mp. [mp = title, abstract, heading word, drug trade name, original title, device manufacturer, drug manufacturer, device trade name, keyword, floating subheading word, candidate term word]

(policy or policies).mp. [mp = title, abstract, heading word, drug trade name, original title, device manufacturer, drug manufacturer, device trade name, keyword, floating subheading word, candidate term word]

28-Practice Guidelines as Topic/

29-(best?practice? or best practice?).mp. [mp = title, abstract, heading word, drug trade name, original title, device manufacturer, drug manufacturer, device trade name, keyword, floating subheading word, candidate term word]

30-or/23–29

31-“diffusion of innovation”/

32-Clinical Decision-Making/

33-“Diagnostic Techniques and Procedures”/

34- Decision Making, Organizational/

35- information dissemination/

36- “Access to Information”/

37-Knowledge Management/

38-Translational Medical Research/

39-(Translational adj (medic* or research)).mp. [mp = title, abstract, heading word, drug trade name, original title, device manufacturer, drug manufacturer, device trade name, keyword, floating subheading word, candidate term word]

40-(Clinical decision making or clinical decision-making or diagnos* technique? or diagnos* procedure? or “barrier? to conduct*” or “barrier? to research conduct*” or “barrier? to application?” or “barrier? to research application?” or research barrier? or conduct barrier? or application barrier? or “facilitator? to conduct*” or “facilitator? to research conduct*” or “facilitator? to application?” or “facilitator? to research application?” or research facilitator? or conduct facilitator? or application facilitator? or “application of knowledge” or appl* knowledge).mp. [mp = title, abstract, heading word, drug trade name, original title, device manufacturer, drug manufacturer, device trade name, keyword, floating subheading word, candidate term word]

41- ((Data or datum or information or knowledge or research* or innovation?) adj2 (share? or sharing or disseminat* or distribut* or manag* or diffus* or translat* or transfer* or utilize or utilise or utili?ation or synthes* or implement* or exchang* or access* or barrier? or facilitat* or select* or tailor* or attitude?)).mp. [mp = title, abstract, heading word, drug trade name, original title, device manufacturer, drug manufacturer, device trade name, keyword, floating subheading word, candidate term word]

42-or/31–41

43–22 and 30 and 42

44-limit 43 to (english language and humans)

## Abbreviations

CAM: Complementary and alternative medicine
EBP: Evidence-based practice
EBM: Evidence-based medicine
TRI: Traditional research-intensive
PBRN: Practice-based research network
EIP: Evidence-informed practice
oBSTACLES: BarrierS To the Application and Conduct of rEsearch
